# Current-Carrying Wear Behavior of the Laser-Alloyed Al/W Composite Layer Under Different Currents

**DOI:** 10.3390/mi16050523

**Published:** 2025-04-29

**Authors:** Heng Zhang, Bai Li, Yulong Zhu, Congwen Tang, Pengfei Sun, Tao Lai, Dengzhi Wang

**Affiliations:** 1 School of Optical and Electronic Information, Huazhong University of Science and Technology, Wuhan 430074, China; 2 National Key Laboratory of Electromagnetic Energy Technology, Wuhan 430070, China; 3 Hangzhou Special Equipment Inspection and Research Institute, Hangzhou 310051, China; 4 Wuhan National Laboratory for Optoelectronics, Huazhong University of Science and Technology, Wuhan 430074, Chinadzwang@hust.edu.cn (D.W.)

**Keywords:** aluminum alloy, laser alloying, refractory metal tungsten, current-carrying wear, wear mechanism

## Abstract

The Al/W composite layer was fabricated on the surface of the aluminum alloy using laser alloying technology to enhance the current-carrying wear resistance. Additionally, the current-carrying wear behaviors of the Al/W composite layer and the aluminum alloy substrate were investigated under different currents. The results indicate that the presence of hard phases such as W and Al_4_W in the composite layer significantly enhanced the wear resistance of the material. Specifically, the average friction coefficient of the Al/W composite layer under different currents was reduced by approximately 9.3–35.8% compared to the aluminum alloy substrate, and the wear rate under current-carrying conditions decreased by about 1.9–6.0 times. For the aluminum alloy substrate, adhesive wear is the dominant mechanism under currents ranging from 0 to 60 A. However, as the current increased to 80 A, the severity of arc erosion intensified, and the wear mechanism transitioned to a combination of arc erosion and adhesive wear. In contrast, for the Al/W composite layer, abrasive wear was the dominant wear mechanism in the absence of electrical current. Upon the introduction of the current, the wear mechanism changed to a coupling effect of arc erosion and adhesive wear.

## 1. Introduction

Electromagnetic rail launch is an advanced technology that operates based on the principle of generating electromagnetic forces through the current flowing between the rails and the armature, thus propelling the armature along with the load to achieve the launch [1,2,3]. This technology boasts significant technical advantages, such as high initial velocity, large kinetic energy, and extended range [1,4,5]. The armature, a critical component in this system, is commonly manufactured from aluminum alloy due to its excellent electrical conductivity, high specific strength, and low density [6,7]. During the process of electromagnetic launch, the aluminum alloy armature experiences melting under the effect of electric current. This melting phenomenon facilitates enhanced contact between the armature and the rails while simultaneously reducing the coefficient of friction. However, with the escalation of the launch current, the excessive melting of the aluminum alloy armature may lead to adverse consequences. Primarily, it may result in poor contact between the armature and the rails, triggering arc discharge and subsequent melting ablation on the rail surface [8,9]. Additionally, a significant amount of molten aluminum is directly deposited onto the rail surface. This deposition has detrimental effects on subsequent launches, compromising the accuracy and reliability of electromagnetic rail launches, thereby reducing the lifespan of the electromagnetic rail launch equipment [10]. These issues have also emerged as a significant factor that constrains the development of electromagnetic railguns. Consequently, it is essential to appropriately enhance the current-carrying wear performance of aluminum alloy armatures under extreme conditions.

Over the past few decades, scholars from different countries have conducted extensive research and discovered that fabricating coatings on the surface of armatures and rials is an effective and feasible method [10]. Singer et al. applied a PTFE film to the armature surface and found that the film effectively reduced the adhesion of aluminum [11]. However, this study did not consider the thermal expansion coefficients of the metal and PTFE film, nor other physical properties of PTFE such as electrical conductivity. It is well known that the use of ceramic particles like Al_2_O_3_, B_4_C, and SiC as reinforcing phases to reduce surface wear and adhesion constitutes one of the prominent research directions in the field of aluminum alloy surface reinforcement at present [12]. This approach can effectively prevent excessive ablation of the aluminum alloy armature during the process of electromagnetic rail launch. Nevertheless, these reinforcing phases also exhibit poor electrical conductivity and high hardness characteristics, which have adverse effects on the overall performance of electromagnetic rail launches [13,14,15]. In order to simultaneously achieve the requirements of electrical conductivity and wear resistance, Lu et al. utilized magnetron sputtering technology to deposit refractory metal tungsten (W) films with a higher melting point on the surface of aluminum alloy [16]. The study demonstrated that the friction coefficient was reduced by approximately 45%, with little effect on the electrical conductivity of aluminum alloy. However, the thickness of the film remains a limiting factor due to its relatively small scale. Based on these findings, the preparation of refractory metal coating on the surface of the armatures and rials, along with proper control over their melting processes, can not only effectively ensure the contact effect between the armatures and rials but also enhance the electromagnetic launching performance.

Laser alloying technology represents an advanced manufacturing technology [17]. The composite layer fabricated using this technology demonstrates several distinct advantages, such as a small heat-affected zone, high bonding strength, a dense structure, and a moderate thickness [18,19]. Refractory metal tungsten (W), with its moderate hardness, low specific resistance, high melting point, and excellent wear resistance [20,21], can be regarded as an ideal material for preparing conductive and wear-resistant coating.

In this study, the Al/W composite layer on the aluminum alloy was prepared using laser alloying technology. The microstructure, microhardness, and electric conductivity of the composite layer were investigated. Furthermore, the current-carrying wear behaviors of both the Al/W composite layer and the aluminum alloy substrate in contact with CuCrZr copper under different currents were assessed, and the associated wear mechanisms were discussed.

## 2. Materials and Methods

### 2.1. Composite Layer Preparation

In this study, the base material employed was 7075 aluminum alloy, whose chemical composition is shown in Table 1. The material used for laser alloying was polygonal pure W powder (purity > 99.9%, particle size 15–35 µm). Before laser alloying, the aluminum alloy substrate was sandblasted and subsequently cleaned with ethanol. To remove moisture, the pure W powders were placed in the 100 °C drying oven for 60 min.

**Figure 1:**
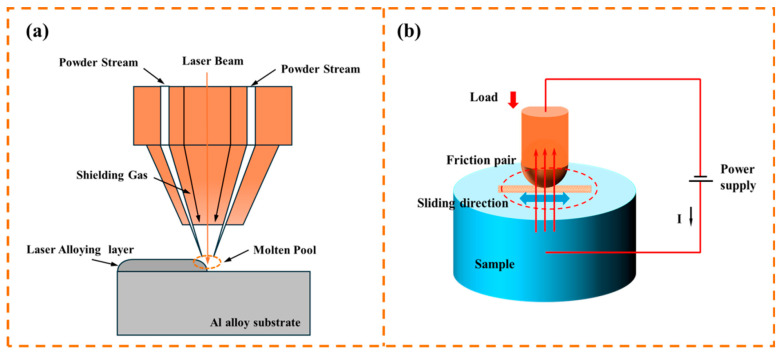
Schematic diagram of (**a**) the laser alloying process and (**b**) current-carrying wear.

The laser alloying process was carried out with the continuous-wave laser (IPG, YLR-6000, IPG Photonics, Oxford, MA, USA) with a wavelength of 1064 nm. The optimized process parameters were as follows: a laser spot diameter of 4 mm, a laser power of 3600 W, a powder feeding rate of 8 g/min, and a laser scanning speed of 650 mm/min. Pure argon gas (purity > 99.99%) was employed as a coaxial protective gas, and the schematic diagram of the laser alloying process is shown in Figure 1a.

The microstructure and element distribution of the samples were observed using SEM-EDS and EPMA (EPMA-8050 G, Shimadzu, Japan). The phase composition of the composite layer was analyzed using XRD (X’ Pert3 Powder, Malvern Panalytical, The Netherlands). During the XRD experiment, a Cu Kα radiation source was utilized for scanning from 20° to 120°. An HVS-1000 microhardness tester was employed to measure the microhardness of the sample with a load of 2 N. Additionally, the electrical conductivity of the sample was tested with a Sigma 2008 A1 digital eddy current conductivity meter.

### 2.2. Tribological Test

Friction and wear tests were conducted using a reciprocating friction machine (MPT-100). The schematic diagram of the testing machine is presented in Figure 1b. The reciprocating wear distance was 15 mm, and the diameter of the CuCrZr copper alloy ball used in the experiments was 6 mm. The chemical composition of CuCrZr copper alloy ball is shown in Table 2. During the experiments, a load of 5 N was applied for a duration of 10 min under various electrical current settings, including 0 A, 20 A, 40 A, 60 A, and 80 A. To ensure reliability and repeatability, each experimental condition was tested three times. After each wear cycle, the CuCrZr copper alloy balls were replaced to facilitate subsequent analysis and maintain consistent testing conditions. The average friction coefficient was the average value of the time-average values taken from three repeated experiments.

After the sliding test, the wear morphology of the worn samples was examined with a laser confocal microscope (VK-X250K), and the wear profiles and wear volumes were calculated using the built-in VK-H1XMC muti-file analysis software. The wear depth values were determined by taking 50 cross-sectional profile curves of the wear scar of each group of experiments and averaging them. The wear rate was calculated based on the following formula:

(1) $$W=\frac{V}{FL}$$

where W is the wear rate (mm^3^ N^−1^ m^−1^), V is the wear volume (mm^3^), F is the normal load(N), and the L is the sliding distance (m). Scanning electron microscopy was used to observe the worn surfaces, and the distribution of the elements was analyzed with an energy spectrometer.

## 3. Results

### 3.1. Phases and Microstructure of the Al/W Composite Layer

**Figure 2:**
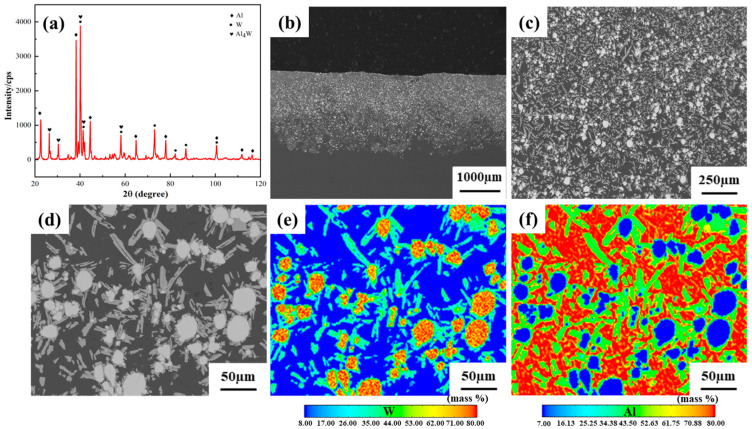
(**a**) XRD patterns, (**b**–**d**) SEM images, and (**e**,**f**) EPMA mappings of the Al/W composite layer.

The XRD patterns of the Al/W composite layer are presented in Figure 2a. The results indicate that the composite layer primarily consists of Al, W, and Al_4_W phases.

Further microstructural characterization is provided by SEM images and EPMA mappings, as shown in Figure 2b–f. The SEM images in Figure 2b,c demonstrate a uniform distribution of W and Al_4_W phases within the Al/W composite layer. In addition, the composite layer exhibits metallurgical bonding with the aluminum alloy substrate, indicating a strong bond between the layer and the aluminum alloy substrate. According to the EPMA results illustrated in Figure 2e,f, it can be inferred that the white block structures correspond to W phases, and the light gray structures represent Al_4_W phases. These findings are consistent with our previous research [22].

### 3.2. Microhardness and Electrical Conductivity

The results of the XRD and SEM revealed that the Al/W composite layer exhibited a three-phase structure comprising Al, W, and Al_4_W phases. To characterize the mechanical properties of these distinct phases, microhardness measurements were conducted separately. The average microhardness values of the W and Al_4_W phases, the Al phase were determined to be 183.2 HV and 136.2 HV, respectively. Notably, the W and Al_4_W phases exhibited higher microhardness, exceeding that of the Al phase by 47.0 HV. Electrical conductivity measurements demonstrated that the aluminum alloy substrate and the Al/W composite layer had electrical conductivities of 34.9%IACS and 10.9%IACS (International Annealed Copper Standard), respectively. This indicates that the electrical conductivity of the aluminum alloy substrate was approximately three times greater than that of the Al/W composite layer. Previous studies have demonstrated that the addition of an alloying element increases electron scattering and thus decreases electrical conductivity [23].

### 3.3. Tribological Properties

#### 3.3.1. Friction Coefficient

**Figure 3:**
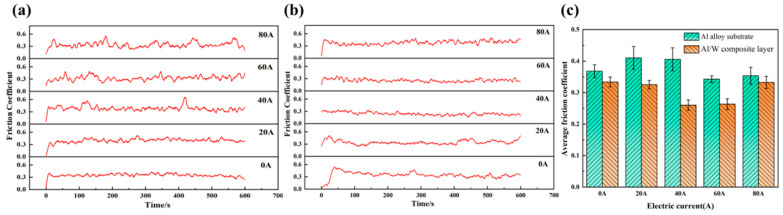
Friction coefficient curves of (**a**) the aluminum alloy substrate, (**b**) the Al/W composite layer, and (**c**) the average friction coefficient under different currents.

Figure 3a,b show the friction coefficient curves of both the aluminum alloy substrate and the Al/W composite layer under different currents. It can be observed that the friction coefficient curves of both materials display slight fluctuations in the absence of current. However, current applications induced progressive destabilization. Notably, the amplitude of fluctuations was substantially more pronounced in the aluminum alloy substrate compared to the Al/W composite layer, demonstrating the enhanced interfacial stability achieved through tungsten reinforcement.

As illustrated in Figure 3c, the average friction coefficient of the aluminum alloy substrate ranged from 0.34 to 0.41 at different currents. In contrast, the corresponding values for the Al/W composite layer ranged from 0.26 to 0.33, representing a reduction of approximately 9.3% to 35.8% relative to the aluminum alloy substrate. With increasing current, the average friction coefficient of the aluminum alloy substrate initially increased, then decreased, and increased again at the current of 80 A. Conversely, the average friction coefficient of the Al/W composite layer exhibited a trend characterized by an initial decrease followed by an increase.

#### 3.3.2. Wear Rate

**Figure 4:**
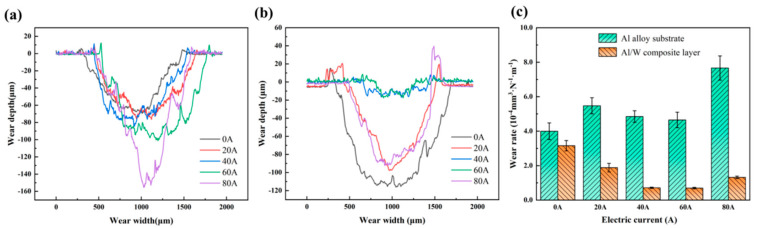
Cross-sectional profiles of the wear scars of (**a**) the aluminum alloy substrate, (**b**) the Al/W composite layer, and (**c**) the wear rate under different currents.

As shown in Figure 4a, the wear scar depth of the aluminum alloy substrate increased with rising current, ranging from 63 μm at 0 A to approximately 152 μm at 80 A. Correspondingly, the wear rate exhibited a minimum value of 3.9 × 10^−4^ mm^3^ N^−1^ m^−1^ at 0 A and showed a trend of an initial increase, followed by a decrease, before rising again at higher current levels (Figure 4c). In contrast, as depicted in Figure 4b, the Al/W composite layer displayed a different wear behavior. The wear depth initially decreased from 118 μm at 0 A to a minimum of 15 μm at 40 A, subsequently increasing to reach a value of 85 μm at 80 A, thus exhibiting a trend of an initial decrease followed by an increase with rising current. In parallel, the wear rate of the Al/W composite layer displayed a maximum wear rate of 3.1 × 10^−4^ mm^3^ N^−1^ m^−1^ at 0 A. Notably, the wear rate reached the minimum value of 0.7 × 10^−4^ mm^3^ N^−1^ m^−1^ at the current of 40 A, representing a 77.4% reduction compared to the 0 A condition and a reduction by six times compared to the aluminum alloy substrate. This reduction in wear rate, combined with the average friction coefficient, demonstrates the improved current-carrying wear resistance of the Al/W composite layer.

#### 3.3.3. Wear Morphology

(1) Wear scar morphology

**Figure 5:**
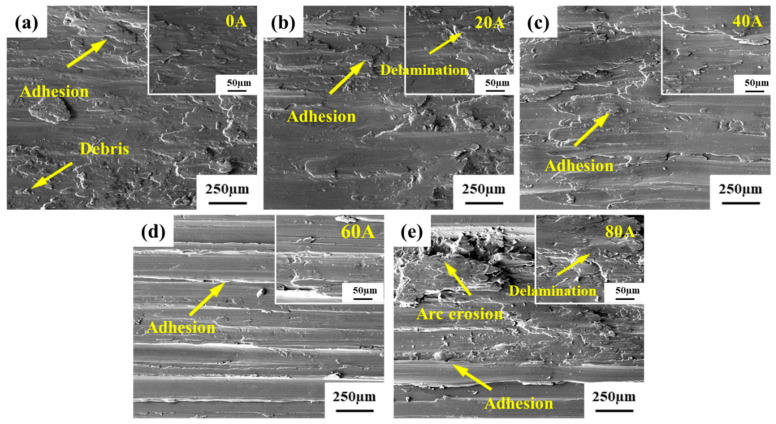
SEM images of the wear surface of the aluminum alloy substrate under different currents: (**a**) 0 A; (**b**) 20 A; (**c**) 40 A; (**d**) 60 A; (**e**) 80 A.

Figure 5 illustrates the morphology of the wear surface of the aluminum alloy substrate under different currents. Under current-free conditions (0 A), the wear surface exhibited extensive flaky adhesive patches accompanied by localized tearing features, which are characteristic manifestations of adhesive wear. Upon the application of electrical current, Ohmic heating effects induced the softening of the aluminum alloy substrate, leading to a progressively smoother wear surface. Additionally, the adhesive layers expanded into larger sheets across the surface. As the current further increased to 60 A, continuous parallel and regular bands aligned with the sliding direction emerged on the wear surface, characterized by the elimination of tear marks and enhanced surface integrity. It was found that adhesive wear remained the dominant wear mechanism under currents of 20–60 A. However, a transition was observed at the current of 80 A, where metal spattering began to occur at peripheral regions of wear scars, indicating that arc erosion phenomena became markedly intensified. At the same time, there was an increase in tearing marks on the wear surface compared to 60 A conditions, along with expanded delamination areas and deteriorated surface roughness. The primary wear mechanism changed to a coupling effect of adhesive wear and arc erosion.

**Figure 6:**
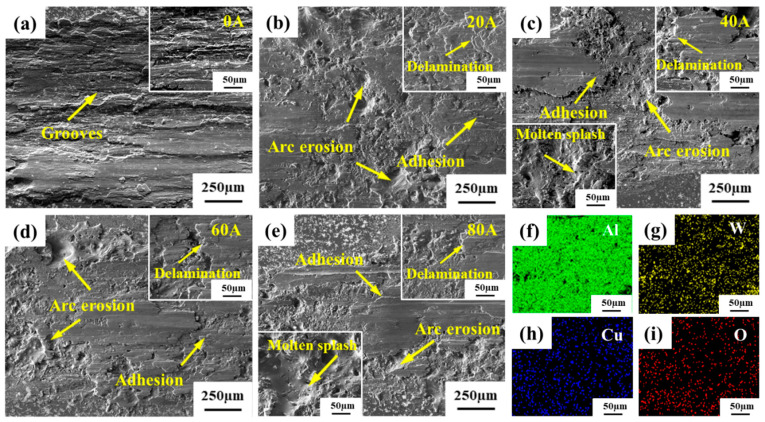
SEM images and EDS mappings of the wear surface of the Al/W composite layer under different currents: (**a**) 0 A; (**b**) 20 A; (**c**) 40 A; (**d**) 60 A; (**e**) 80 A; (**f**–**i**) the EDS mappings at 0 A.

Figure 6 presents the morphology of the wear surface of the Al/W composite layer under different currents. In the absence of electrical current, the wear surface exhibits deep and wide grooves, characteristic of typical abrasive wear. Surrounding these grooves are bright white particles, which are primarily fragmented tungsten (W) generated during the wear process according to EDS analysis presented in Figure 6g. As the current increased to 20 A, distinct arc ablation pits emerged on the wear surface. Compared to the no-current condition, the dimensions of the grooves were significantly reduced, leading to a smoother surface topography and fewer bright white particles along the periphery of the wear scars. Notably, adhesion phenomena occurred on the wear surface. When further increasing the current to 40 A, adhesion effects became more pronounced alongside intensified arc erosion characteristics marked by increased molten metal splattering and broader droplet dispersion across the surface. At the current level of 60 A, tearing phenomena were observed concurrently with adhesion on the wear surface. A multitude of ablation pits with larger sizes than those at 40 A appeared at the edges. When the current was 80 A, the increased current resulted in exacerbated arc erosion characteristics on the wear surface, with enlarged ablation pit dimensions and broader spatial distribution. At the same time, the wear surface roughness intensified, featuring a complex interplay of adhesion layers and tearing phenomena. Based on the above, it can be concluded that the dominant wear mechanisms affecting the Al/W composite layer under current-carrying conditions were the coupling of arc erosion and adhesive wear. Moreover, as the current increased, the arc erosion effect became more significant.

(2) Wear surfaces’ cross-sectional morphology

**Figure 7:**
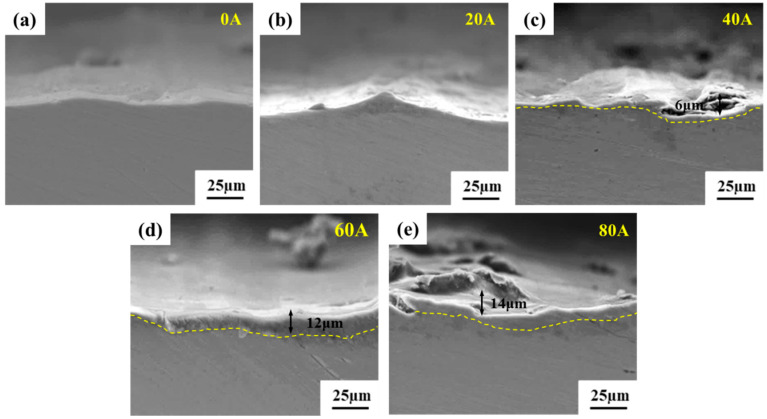
SEM images of the cross-sectional morphology of the aluminum alloy substrate wear specimens under different currents: (**a**) 0 A; (**b**) 20 A; (**c**) 40 A; (**d**) 60 A; (**e**) 80 A.

Figure 7 shows the cross-sectional morphology of the aluminum alloy substrate wear specimens under different currents. At 0 A and 20 A, no obvious changes were observed in the cross-section of the wear specimens. However, as the applied current increased, a distinct melting layer emerged at 40 A, with an estimated thickness of approximately 6 μm. Notably, with further escalation of the current, the thickness of this melting layer progressively increased, reaching approximately 14 μm at 80 A.

**Figure 8:**
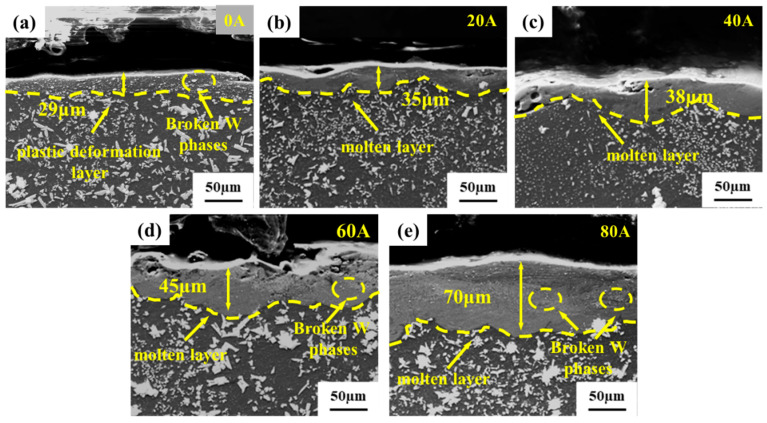
SEM images of the cross-sectional morphology of the Al/W composite layer wear specimens under different currents: (**a**) 0 A; (**b**) 20 A; (**c**) 40 A; (**d**) 60 A; (**e**) 80 A.

Figure 8 illustrates the cross-sectional morphology of the Al/W composite layer wear specimens under different currents, revealing distinct microstructural characteristics in the shallow surface layer between the aluminum alloy substrate and the composite layer. Without the application of current, a plastic deformation layer with a thickness of approximately 29 μm was observed on the surface of the Al/W composite layer. Within this layer, W particles and Al_4_W phases were crushed under the combined action of frictional force and normal pressure. With the introduction of electrical current, a molten reaction layer emerged on the wear surface’s cross-section. This transformation is attributed to the current-induced melting of the Al matrix within the composite layer, which facilitates the dissolution of W and Al_4_W into the molten aluminum. The thickness of the melting reaction layer exhibited a clear dependence on the applied current, measuring approximately 35 μm at 20 A and increasing to approximately 70 μm at 80 A. Furthermore, the microstructural characterization of the wear specimen’s cross-section demonstrates a notable difference in W particle morphology: under current-free conditions, the crushed W particles maintained relatively larger dimensions, whereas under current-carrying conditions, the dissolved W particles exhibited finer granularity and more homogeneous distribution. These observations indicate that the applied current facilitated both the melting of aluminum and the dissolution of tungsten within the composite layer, ultimately leading to the formation of a lubricating liquid aluminum film on the wear surface. This film effectively reduced abrasive wear between the composite layer and the copper alloy ball, thereby enhancing the tribological performance of the materials.

## 4. Discussion

During the wear process, the contact between the friction pairs is achieved through the interconnection of uneven micro-asperities on their surfaces [24]. In the absence of electric current, these micro-asperities only bear the effect of mechanical friction. However, after the current is introduced, it flows through the conductive points formed by these micro-asperities across the friction interface, resulting in a wear process driven by the combined effects of both mechanical friction and electrical current. Notably, the dynamic changes in surface-contacting micro-asperities during frictional sliding induce continuous reconstruction of the conductive contact points, leading to significant fluctuations in contact resistance and voltage. When the voltage surpasses the critical ionization threshold, localized gas breakdown occurs, accompanied by arc discharge. This discharge process releases substantial thermal and photonic energy [25], which subsequently influences the wear behavior. These complex thermal coupling effects result in different wear morphologies on the wear surface. To explain the wear mechanisms in detail, the wear behaviors of the aluminum alloy substrate and the Al/W composite layer under different currents will be systematically analyzed in the next section.

### 4.1. Aluminum Alloy Substrate

In the absence of current, the primary heat source at the contact surface originates from frictional heating during the wear process. According to the Archard model [26], the relationship between the wear volume (V) and hardness (*H*) of material can be expressed as follows:

(2) $$V=k_{a}\frac{FL}{H}$$

where $k_{a}$ is the dimensionless wear coefficient, *F* and *L*, respectively, represent the applied load and the sliding distance. The above formula indicates that the wear volume is inversely proportional to the hardness, suggesting that materials with greater hardness have stronger resistance to deformation and better wear resistance. The relatively low interfacial temperature in this scenario results in limited thermal softening of the aluminum alloy substrate, thereby maintaining sufficient hardness to resist plastic deformation. Therefore, the wear rate of the aluminum alloy substrate is minimized in the absence of current, with adhesive wear being the dominant mechanism.

**Figure 9:**
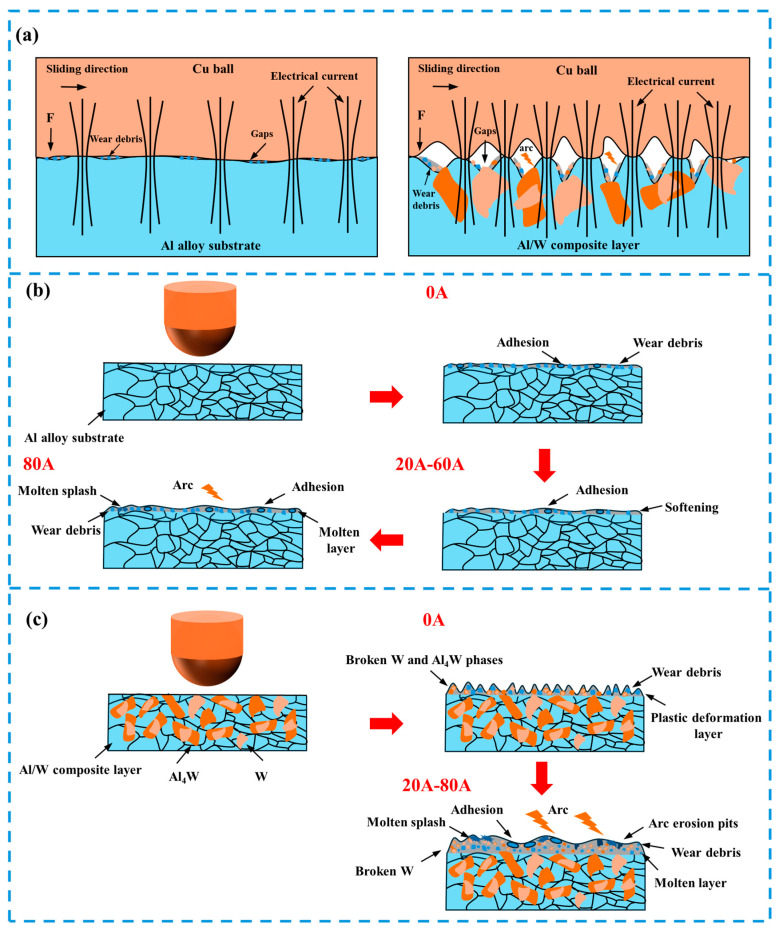
(**a**) Current-carrying wear contact interface; schematic diagram of wear mechanism of (**b**) the aluminum alloy substrate and (**c**) the Al/W composite layer.

When an electrical current is applied, the thermal dynamics at the friction interface become more complex, incorporating both frictional heat and Joule heating. The significant increase in total heat exacerbates the softening of the aluminum alloy substrate surface, weakening its resistance to friction. This intensifies adhesive wear and increases the number of tear points during relative sliding. Furthermore, the micro-asperities at the interface between the aluminum alloy substrate and the copper alloy ball expand and coalesce, forming larger planar contact areas. Notably, there is no heterophase in the aluminum alloy (Figure 9a). Consequently, the current conduction state between the friction pairs is stable. This explains why no obvious arc erosion marks were observed on the wear surface of the aluminum alloy substrate at low currents. Instead, only an aggravated plastic deformation of the surface layer occurred under the effect of combined heat and intensified mechanical wear, leading to an increased wear rate, with adhesive wear remaining the primary mechanism. Additionally, when the contact interface temperature reaches the melting point of the aluminum alloy, a liquid molten layer forms on the surface of the aluminum alloy substrate, which can act as a lubricant and reduce the friction coefficient and wear rate [27]. Therefore, as the test current increased from 20 A to 60 A, the friction coefficient and wear rate of the aluminum alloy substrate showed a decreasing trend.

However, when the current increased to 80 A, the arc erosion effect significantly intensified, with obvious arc erosion marks appearing on the wear surface, leading to an increase in wear. The friction coefficient and wear rate increased accordingly. Moreover, the wear mechanism transitioned to a coupling effect of adhesive wear and arc erosion. The schematic diagram of wear mechanisms is shown in Figure 9b.

### 4.2. Al/W Composite Layer

In the Al/W composite layer, the hardness of the W and Al_4_W phases was higher than that of the Al phases. Consequently, the softer Al phases were preferentially worn away during the wear process without currents, while the harder W and Al_4_W phases remained embedded within the composite layer. These hard phases acted as reinforcing components, effectively inhibiting wear damage progression and reducing both the friction coefficient and the wear rate. However, as the wear process progressed, these hard phases gradually cracked and flaked off under the repeated action of the frictional force and normal pressure, generating abrasive particles that led to three-body abrasive wear. As a result, the wear rate of the Al/W composite layer was maximum under current-free conditions.

The introduction of electrical current significantly altered the wear dynamics. The preferential wear of the softer Al phase between the harder W and Al_4_W phases resulted in microscopic gaps (Figure 9a). When the electric field intensity exceeds a certain threshold, arc discharge occurs. The field emission current density can be determined according to the Fowler–Nordheim equation [28]:

(3) $$I_{c}=\frac{1.541\times 10^{-6}E^{2}}{\Phi }exp\left[-6.831\times 10^{9}\frac{\Phi^{\frac{3}{2}}}{E}\theta (y)\right]$$

where $I_{c}$ is the field emission current density (A/m^2^), E is the applied electric field strength (V/m), θ(y) is the Nordheim elliptic function, and Φ is the work function (eV), which is the least amount of energy required for electrons to leave the surface potential. While maintaining a constant electric field density, the electron emission current increases significantly when the work function decreases. Consequently, electric-arc erosion tends to occur preferentially on the Al phase with a low work function (W: ~4.67 eV, Al: ~4.28 eV) [29,30] during current-carrying wear.

The instantaneous temperature during arc discharge can reach 3000–5000 K [31]. Under the action of the arc, aluminum on the surface of the composite layer melts rapidly, while the harder W and Al_4_W dissolve in the liquid aluminum and form a liquid film together. The existence of the liquid film can significantly reduce the friction coefficient and wear rate. In addition, the dissolution of the hard phases W and Al_4_W also eliminates the abrasive wear phenomenon of the composite layer. Therefore, under the current-carrying conditions, the dominant wear mechanisms of the composite layer transitioned to a coupling effect of adhesive wear and arc erosion. At lower currents, due to the lubricating effect of the liquid film, a decrease was observed in both the friction coefficient and wear rate. However, when the current increased to 80 A, intensified arc erosion led to an increase in the friction coefficient and wear rate, though the wear mechanism remained a combination of arc erosion and adhesive wear. Based on the above, it can be inferred that W and Al_4_W phases act as reinforcing components, effectively inhibiting wear damage progression and reducing the wear rate. The schematic diagram of wear mechanisms is shown in Figure 9c.

## 5. Conclusions

In this work, the Al/W composite layer on the surface of the aluminum alloy was prepared using laser alloying technology. The wear behaviors of the Al/W composite layer and aluminum alloy substrate under different currents were investigated and analyzed. However, the current-carrying wear experiments under higher current conditions were not conducted. At present, this study can only provide some directional guidance for research on the current-carrying wear damage of the armatures and tracks. The primary conclusions are as follows:

Compared to the aluminum alloy substrate, the average friction coefficient of the Al/W composite layer under different currents was reduced by approximately 9.3–35.8%, and the wear rate under current-carrying conditions decreased by about 1.9–6.0 times. These findings indicate that the Al/W composite layer exhibited improved current-carrying wear resistance.

The friction coefficient and wear rate of the aluminum alloy substrate both showed a trend of an initial increase, then a decrease with rising current, followed by another increase at higher currents. Within the current range of 0–60 A, the wear mechanism was mainly adhesive wear. However, as the current increased to 80 A, the severity of arc erosion intensified, leading to aggravated wear damage. The wear mechanism transitioned to a coupling effect of arc erosion and adhesive wear.

The friction coefficient and wear rate of the Al/W composite layer both exhibited an initial decrease followed by an increase as the current increased. Under the current of 0 A, the primary wear mechanism was abrasive wear, but after introducing the current, it changed to a coupling effect of arc erosion and adhesive wear.

## Figures and Tables

**Table 1:** Chemical composition of 7075 aluminum alloy substrate (wt. %).

Element	Mg	Zn	Cu	Si	Ti	Mn	Ti	Cr	Fe	Al
Wt. %	2.1–2.9	5.1–6.1	1.2–2.0	0.40	0.20	0.30	0.20	0.18–0.28	0.50	Bal

**Table 2:** Chemical composition of CuCrZr copper alloy ball.

Element	Al	Mg	Cr	Zr	Si	Fe	P	Cu
Wt. %	2.1–2.9	5.1–6.1	1.2–2.0	0.40	0.20	0.30	0.20	Bal

## Data Availability

Data are contained within this article.
